# Exploring the effect of plant substrates on bacterial community structure in termite fungus-combs

**DOI:** 10.1371/journal.pone.0232329

**Published:** 2020-05-01

**Authors:** Shiyou Liang, Chengpan Wang, Farhan Ahmad, Xuejie Yin, Yin Hu, Jianchu Mo

**Affiliations:** 1 Ministry of Agriculture Key Laboratory of Agricultural Entomology, Institute of Insect Sciences, Zhejiang University, Hangzhou, Zhejiang, China; 2 Entomology Section, Central Cotton Research Institute Sakrand, Shaheed Benazirabad, Sindh, Pakistan; 3 National Termite Control Center, Hangzhou, Zhejiang, China; Osmania University, INDIA

## Abstract

Fungus-cultivating termites are successful herbivores largely rely on the external symbiotic fungus-combs to decompose plant polysaccharides. The comb harbors both fungi and bacteria. However, the complementary roles and functions of the bacteria are out of the box. To this purpose, we look into different decomposition stages of fungus-combs using high-throughput sequencing of the 16S rRNA gene to examine bacterial community structure. We also explored the bacterial response to physicochemical indexes (such as moisture, ash content and organic matter) and plant substrates (leaves or branches or mix food). Some specific families such as Lachnospiraceae, Ruminococcaceae, and Peptostreptococcaceae may be involved in lignocellulose degradation, whereas Burkholderiaceae may be associated with aromatic compounds degradation. We observed that as the comb mature there is a shift of community composition which may be an adjustment of specific bacteria to deal with different lignocellulosic material. Our results indicated that threshold amount of physicochemical indexes are beneficial for bacterial diversity but too high moisture, low organic matter and high ash content may reduce their diversity. Furthermore, the average highest bacterial diversity was recorded from the comb built by branches followed by mix food and leaves. Besides, this study could help in the use of bacteria from the comb of fungus-cultivating termites in forestry and agricultural residues making them easier to digest as fodder.

## Introduction

Herbivorous insects obtain energy and nutrients from their host plants. However, lignocellulose in plants biomass is highly recalcitrant to enzymes attack. To overcome this barrier, these insects have evolved specific feeding tactics [1] or digestive capacities such as masticating organs, gut structures, digestive enzymes, and symbiotic systems [2]. Another challenge is a wide variety of constitutive, or induced defense compounds. These chemical defenses can act as repellents, or antinutritives, or toxics, or attract predators [1, 3–5]. The herbivores have also adapted some strategies against these compounds. For example, the desensitization of insect proteins targeted by plant defenses or the modification of ingested plant chemicals [3]. The capacity of herbivores to break these two obstacles shape them to be specialists or generalists.

**Fig 1:**
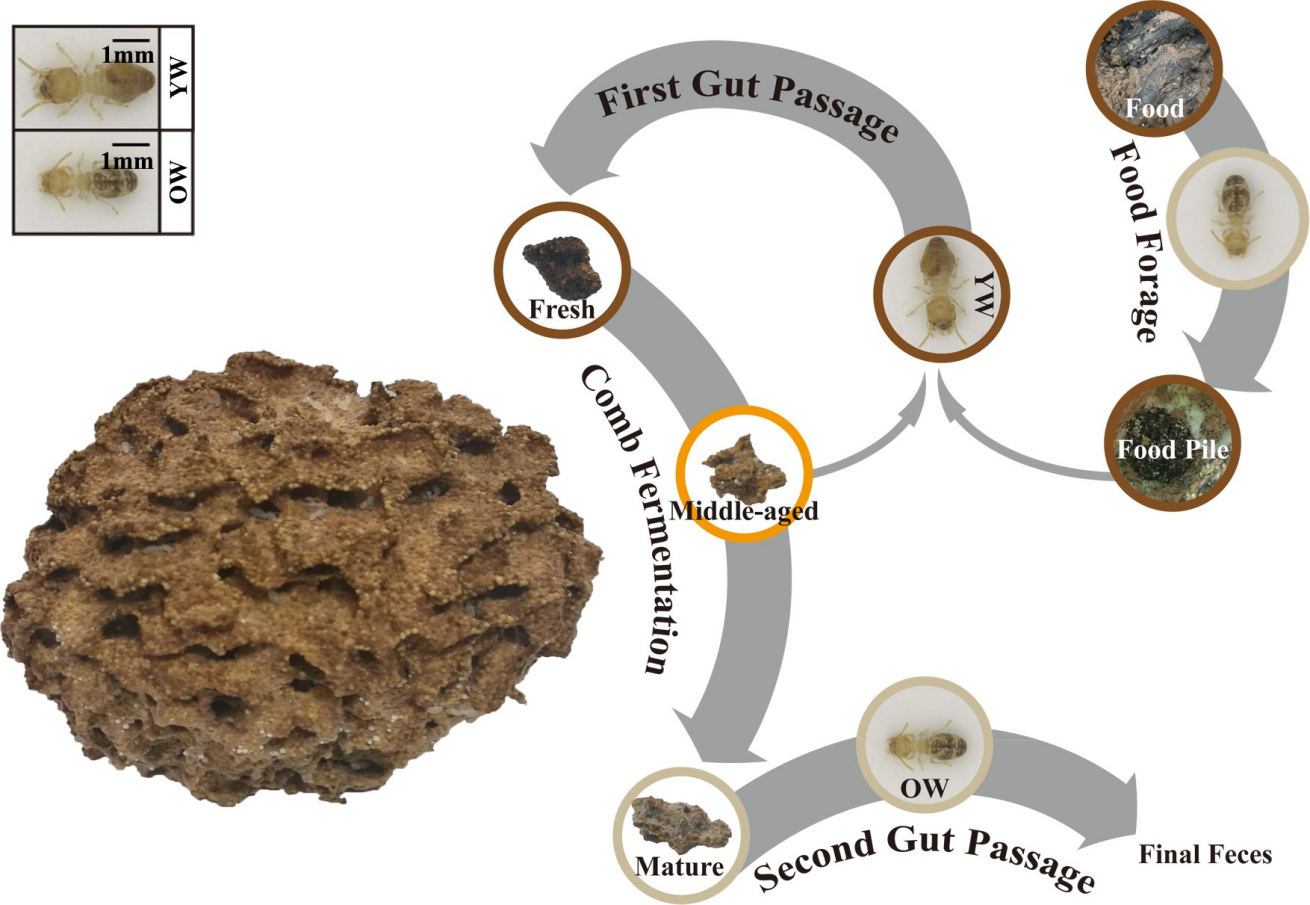
The food processing pathway in fungus-cultivating termite (*O*. *formosanus*). Food forage: old workers collect the food from outside environment and pile in the colony; First gut passage: young workers ingest the foraged material along with nodules (conidia) from middle-aged fungus-comb and excrete primary feces on the top surface of the comb to establish fresh comb; Comb fermentation: fungus grows on the fresh comb, degrade it efficiently to convert it into middle-aged comb, where new nodules are produced [12, 13]. The comb turns to mature in 45 days [12]; Second gut passage: old workers ingest mature comb and excrete final feces. YW, young worker; OW, old worker.

Fungus-cultivating termites (Blattodea, Termitidae, Macrotermitinae) are successful herbivores, feed on various plant materials such as dry grass, leaf litter, root and ruminant dung [6, 7]. They contribute to more than 90% of plant biomass in arid tropics [8] through the efficient degradation of highly recalcitrant lignocellulosic materials. To this purpose, they cultivate special basidiomycete fungi (Agaricomycetes, Lyophyllaceae, *Termitomyces*) in their nests as an external “digestive” system called fungus-combs. The food processing in Macrotermitinae presents a complex age polyethism involving two gut passages [9–11] (Fig 1).

The termite comb not only harbor fungus but also bacteria [14–16]. The lignocellulolytic potential of *Termitomyces* has been demonstrated by enzyme activity assays of culture *in vitro* [17, 18], RNAseq *in situ* [19], and genome sequencing [10]. However, the functions of the bacteria in comb are unclear. In wood-feeding termite, Fibrobacteres, Firmicutes, Bacteroidetes, the TG3 phylum, Elusimicrobia, Spirochaetes are the fiber associated lignocellulolytic bacteria [20–23]. Some studies indicated that bacteria in fungus comb have antimicrobial, polycyclic-degrading and cellulose-degrading activity [24–27]. Otani et al. [15] and Long et al. [16] described the presence of bacterial community in field-collected fungus-combs, but their studies are unable to clarify what kind of community composition are active at different decomposition stages of comb and how do they respond to different plant substrates?

Therefore, we fed fungus-cultivating termite (*Odontotermes formosanus*) with three different kinds of lignocellulose diets such as leaves, branches, and mix food respectively in laboratory and sampled the fungus-combs at different stages of decomposition or ages. Further, we also explored the impact of three physicochemical indexes (moisture, ash content and organic matter) on bacterial diversity in fungus-combs.

## Materials and methods

### Termite colonies feeding and sample collection

Three colonies of *O*. *formosanus* (height ≈ 6–8 cm; diameter ≈ 12–15 cm) harboring king(s) and queen(s) were collected from a forested area in Sanming, Fujian Province, China (117.90° E, 25.53° N). The private land owner gave permission for this collection. The colonies along with fungus-combs were wrapped in plastic film separately and transported to the lab within 12 h of excavation. All colonies were placed separately in plastic chambers (45 × 45 ×30 cm, length × width × depth) containing clay soil obtained from the area where the colonies were collected. The rearing systems were maintained in complete darkness at 26 ± 1°C and > 90% relative humidity. After the colonies became stable in a laboratory, they were allowed to feed on one of the three different kinds of food, including i) leaves or ii) branches of *Quercus palustris*, or iii) mix food collected from Laohe Mountain, Hangzhou, Zhejiang Province, China. All colonies were successful in building new fungus-combs. We divided the whole comb into three layers i) top or fresh comb, ii) middle or middle-aged comb, and iii) bottom or mature comb (Fig 1). The fungus-combs constructed by individual food were collected as described previously by Li et al. [9]. Briefly, dark brown color for fresh, yellowish-brown for middle-aged and gray for mature. For every sample, 3 g was used to measure the physicochemical indexes immediately, and 0.5 g was stored at—80°C for subsequent microbial diversity analysis.

### Measurement of physicochemical indexes

The moisture, ash content and organic matter were measured from each sample. For moisture content measurement, a weighing bottle was washed, dried in the oven and weighed (m_1_). Approximately 0.5 g fungus-comb was put into the bottle and re-weighed (m_2_), dried in an oven at 105°C for 6 hours, and re-weighed (m_3_) [28]. In case of ash content, a ceramic crucible was washed and dried in a muffle furnace at 550°C for 4 hours and weighed (m_4_). Approximately 0.5 g of totally dried fungus-comb was put into the crucible and re-weighed (m_5_), re-dried at 550°C for 4 hours, and re-weighed (m_6_) [29]. The moisture, ash and organic matter content were determined as follows:

$$\text{Moisture content}=\frac{m_{2}-m_{3}}{m_{3}-m_{1}}\times 100\text{\%}$$

$$\text{Ash content}=\frac{m_{6}-m_{4}}{m_{5}-m_{4}}\times 100\text{\%}$$

Organic matter content=100%−Ash content

### DNA extraction

Approximately, 0.15 g of each sample was ground in a 1.5 ml Eppendorf tube with 800 μl Tris-EDTA (100 mM Tris base, 50 mM EDTA, PH 8.0). The mixture was transferred to a 2 ml screw-cap tube and homogenized by bead-beating [30]. After that, the homogenate was mixed with 100 μl 5 mg/ml lysozyme and incubated at 37°C for 20 min. Next, 60 μl 32 mg/ml protease k and 40 μl 25% SDS, were added and incubated at 37°C for 40 min. Then, the lysate was mixed with 167 μl 5 M NaCl and 133 μl 10% CTAB, and incubated at 65°C for 10 min [31]. After the extraction by phenol-chloroform-isoamyl alcohol (25: 24: 1, v: v: v) from the lysate, the crude DNA was purified using a Qiagen DNeasy column following the protocol as described by the company. The purified DNA in the column was eluted by 100 μl AE buffer (Qiagen).

### Illumina sequencing

The bacterial communities in fungus-combs were analyzed using 454 Illumina sequencing. The primer pair 338F (ACT CCT ACG GGA GGC AGC AG) and 806R (GGA CTA CHV GGG TWT CTA AT) [32] were used to amplify the V3—V4 region of bacterial 16S rRNA genes in the respective DNA samples. The amplified products were purified by the AxyPrep DNA kit (AXYGEN, China). The amplicons were sequenced on Illumina MiSeq by Shanghai Personal Biotechnology Inc., China.

### Data analysis

The physicochemical indexes were analyzed through Duncan's new multiple range method using SPSS, v.22.0 (SPSS Inc., Chicago, IL) at α = 0.05. Raw sequences were quality filtered using the QIIME release 1.8.0 software [33] under the stringent conditions (reads ≥ 150 bp, no ambiguous bases, bases mismatched by 5’ primer ≤ 1 and a maximum number of homopolymers ≤ 8). Subsequent removal of chimeras was carried out through UCHIME [34] by uchime3_denovo. After reducing the redundancy of sequences and demarcating OTU (Operational Taxonomic Unit) with a similarity threshold of 97% using UCLUST [35], sequences with the highest abundance of each OTU were selected as the representative sequences of corresponding OTU. Representative sequences of each OTU were aligned and taxonomically classified in the reference database, SILVA [36] using MOTHUR [37]. A subsequent assignment to the termite-specific reference database (DictDb) [38] was performed with a confidence threshold of 60%. OTUs were simplified by removing the OTUs whose abundance was lower than 0.001% of total sequences [39]. QIIME was used to calculate and map the rarefaction curves based on 97% sequence similarity cutoff and calculate diversity indexes of each sample by uclust. The relative abundance of bacteria at different levels were calculated and mapped through R software (http://www.r-project.org). LEfSe (linear discriminant analysis effect size) analysis was performed to identify discriminatory taxa through the online platform, Galaxy (http://huttenhower.sph.harvard.edu/galaxy/). Heatmap of selected bacterial lineages, the constrained corresponding analysis (CCA) of the bacterial community and variation partition analysis for physicochemical indexes were implemented in R software (packages *pheatmap* and *vegan*). The high-quality sequences of the samples have been submitted to the NCBI Sequence Read Archive (BioProject PRJNA604886); for accession numbers, see S5 Table.

## Results

### Effect of diets and fungus-comb ages on the physicochemical indexes

**Fig 2:**
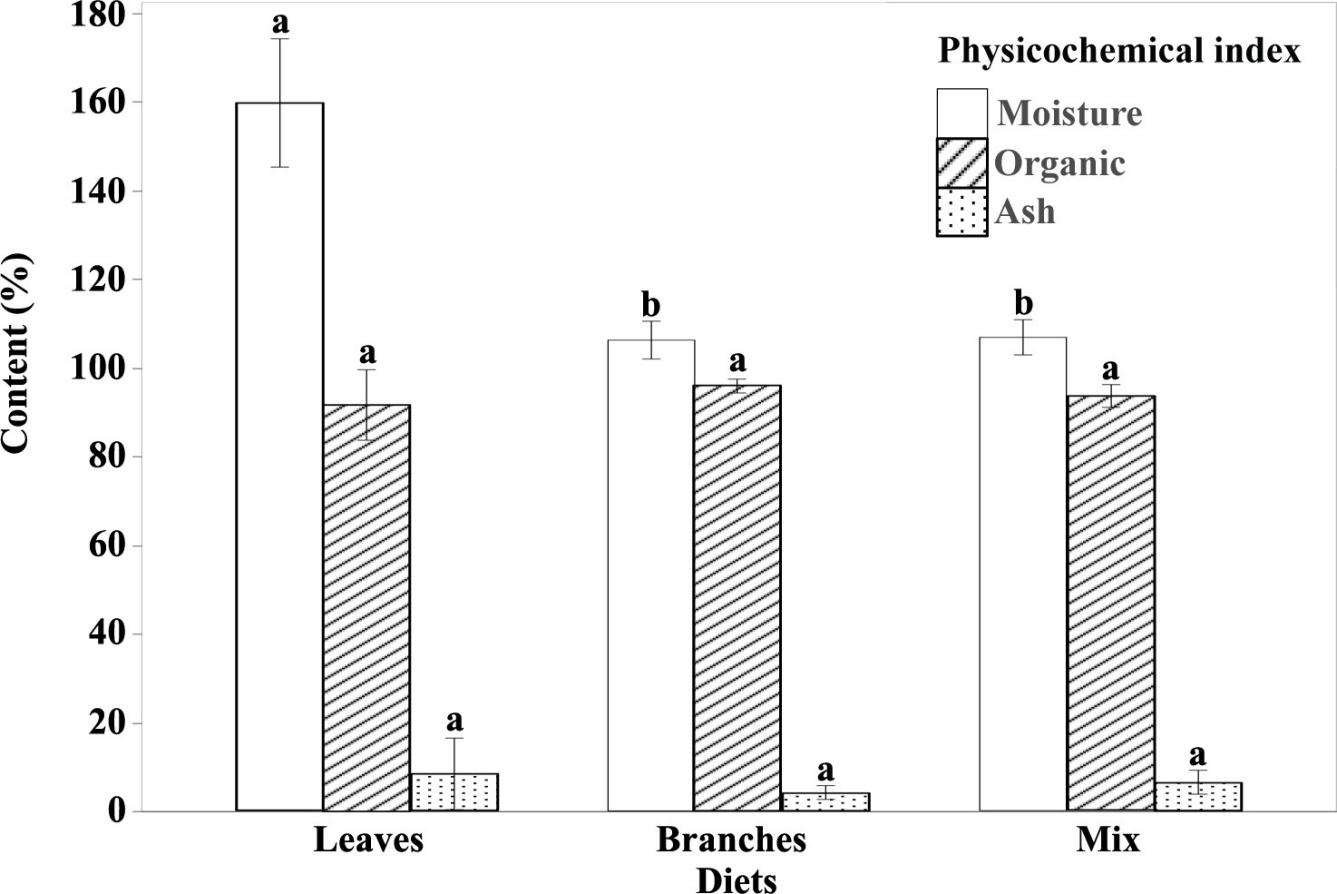
Comparison of the physicochemical indexes among fungus-combs built by different diets. The bar indicates mean±SD (n = 9), bar labeled by different letters of the same index are significantly different as determined by Duncan's new multiple range test (P < 0.05).

The physicochemical indexes including moisture, ash content and organic matter from the fungus-combs built by leaves, branches, and mix food respectively, are summarized in Table 1 and Fig 2. The moisture content at different decomposition stages of the combs i.e., top, middle and bottom was significantly different (d.f = 8, F = 99.927, p<0.001), (d.f = 8, F = 62.247, p<0.001) and (d.f = 8, F = 9.595, p<0.014) among leaves, branches and mixed food, respectively (Table 1). Moreover, comparatively higher moisture was recorded from the comb constructed by leaves followed by mix food and branches.

**Table 1:** Comparison of the physicochemical indexes at different decomposition stages of fungus-combs.

Sample		Moisture content (%)	Ash content (%)	Organic matter (%)	Code
**Leaves**	T	149.80 ± 2.31 a*	4.59 ± 0.01 a	95.41 ± 0.01 a	TL
	M	150.39 ± 1.65 a	1.47 ± 0.73 b	98.53 ± 0.73 b	ML
	B	178.39 ± 4.00 b	18.82 ± 0.99 c	81.18 ± 0.99 c	BL
		d.f = 8; F = 99.927; P < 0.001^$^	d.f = 8; F = 506.157; P < 0.001	d.f = 8; F = 506.187; P < 0.001	
**Branches**	T	101.83 ± 1.11 c	2.50 ± 1.20 ab	97.50 ± 1.20 ab	TB
	M	105.28 ± 1.03 cd	4.96 ± 0.97 b	95.04 ± 0.96 b	MB
	B	111.47 ± 1.08 d	4.98 ± 0.82 b	95.02 ± 0.82 b	BB
		d.f = 8; F = 62.247; P < 0.001	d.f = 8; F = 6.023; P = 0.037	d.f = 8; F = 6.023; P = 0.037	
**Mix**	T	104.82 ± 0.47 c	4.72 ± 0.40 b	95.28 ± 0.40 b	TM
	M	104.27 ± 2.06 c	4.90 ± 0.10 b	95.10 ± 0.10 b	MM
	B	111.49 ± 3.27 d	9.76 ± 1.48 d	90.24 ± 1.48 d	BM
		d.f = 8; F = 9.595; P = 0.014	d.f = 8; F = 31.055; P < 0.001	d.f = 8; F = 31.055; P < 0.001	

*Values represent mean±SD (n = 3), and mean followed by different letters in the same row are significantly different as determined by Duncan's new multiple range test (P < 0.05).

^$^ANOVA analysis of different aged combs of the same diet.

The T, M and B represent the top, middle, and bottom, respectively. Whereas TL, ML, BL, TB, MB, BB, TM, MM, and BM represent top leaves, middle leaves, bottom leaves, top branches, middle branches, bottom branches, top mix, middle mix, and bottom mix, respectively.

Similarly, the ash content was also significantly different at top, middle and bottom parts of the combs (d.f = 8, F = 506.157, p<0.001), (d.f = 8, F = 6.023, p<0.037) and (d.f = 8, F = 31.055, p<0.001) among leaves, branches and mix food combs, respectively (Table 1). But the highest ash content was recorded from leaves built comb followed by mix food and branches.

In case of organic matter content, there was also a significant differences among top, middle and bottom of the comb (d.f = 8, F = 506.187, p<0.001), (d.f = 8, F = 6.023, p<0.037) and (d.f = 8, F = 31.055, p<0.001) constructed by leaves, branches and mix food, respectively (Table 1). Contrary to moisture and ash contents, the maximum organic matter was calculated from branches followed by mix food and leaves combs. Interestingly, the moisture and ash contents were high at the bottom part of all the combs while the organic matter was lower.

### The bacterial community structure among different fungus-combs

**Fig 3:**
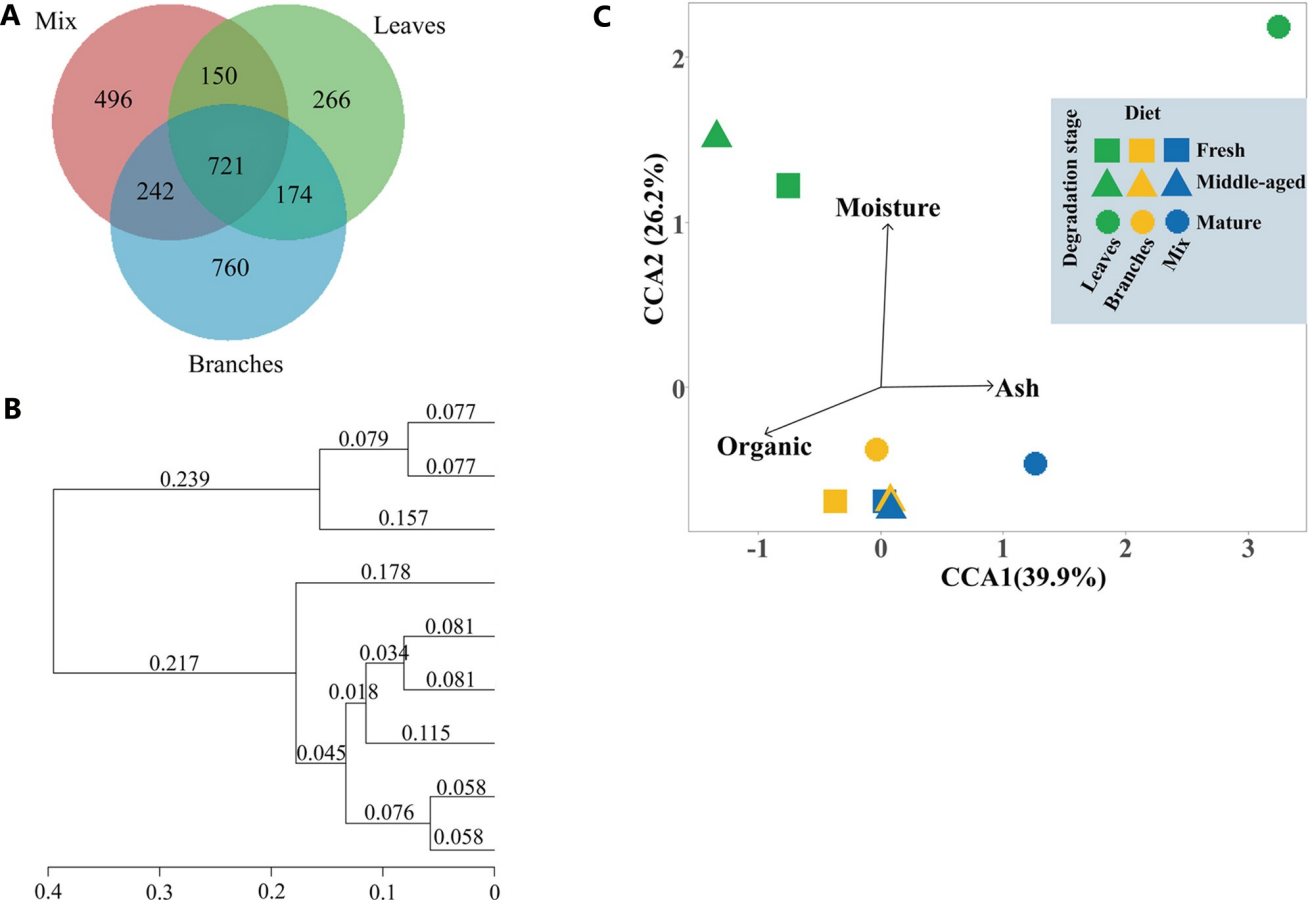
Bacterial community profiles at different decomposition stages of fungus-combs. (A) Common bacteria OTU Venn diagram of fungus-combs built from different foods (OTUs whose abundance lower than 0.001% of total sequences were removed). (B) Community similarities based on weighted Unifrac metric; (C) The constrained corresponding analysis (CCA) of the bacterial community and variation partition analysis for three physicochemical indexes. Whereas TL, ML, BL, TB, MB, BB, TM, MM, and BM described in Table 1. The Denoising stats of OTUs are listed in S6 Table.

The whole comb of mix food, branches and leaves occupied 496, 760 and 266 OTUs, respectively, however, 721 OUTs is common among all (Fig 3A). In case of comb parts, the OTU cluster analysis clearly divided the bacterial community into two groups (Fig 3B). The groups are formed top and middle (group 1) and bottom of all combs (group 2), with bacterial community similarity. The bacterial communities showed similarities at top and middle comb but they were entirely different from bottom combs, irrespective of food (Fig 3B and 3C).

The microbial diversity indexes (Simpson [40] and Shannon [41]), estimated community richness (ACE [42] and Chao1 [43]) (Table 2) described that the average highest diversity was recorded from branches followed by mix food and leaves (Table 2). For comb parts, it increased in the middle and dropped in the bottom comb (Table 2).

**Table 2:** Bacterial diversity indexes at different decomposition stages of fungus-combs.

Sample		Chao1	ACE	Simpson	Shannon
	T	1303.99	1318.62	0.97	7.06
**Leaves**	M	1480.18	1521.59	0.97	7.04
	B	839.90	848.02	0.79	3.49
	Average	1208.02	1229.41	0.91	5.86
	T	1548.21	1524.90	0.98	7.64
**Branches**	M	1823.25	1818.46	0.98	7.34
	B	1180.86	1178.40	0.90	5.56
	Average	1517.44	1507.25	0.95	6.85
	T	1527.56	1560.70	0.95	7.05
**Mix**	M	1574.05	1596.62	0.96	7.08
	B	797.48	783.59	0.86	4.94
	Average	1299.70	1313.64	0.93	6.36

The T, M, and B represent the top, middle, and bottom, respectively.

**Fig 4:**
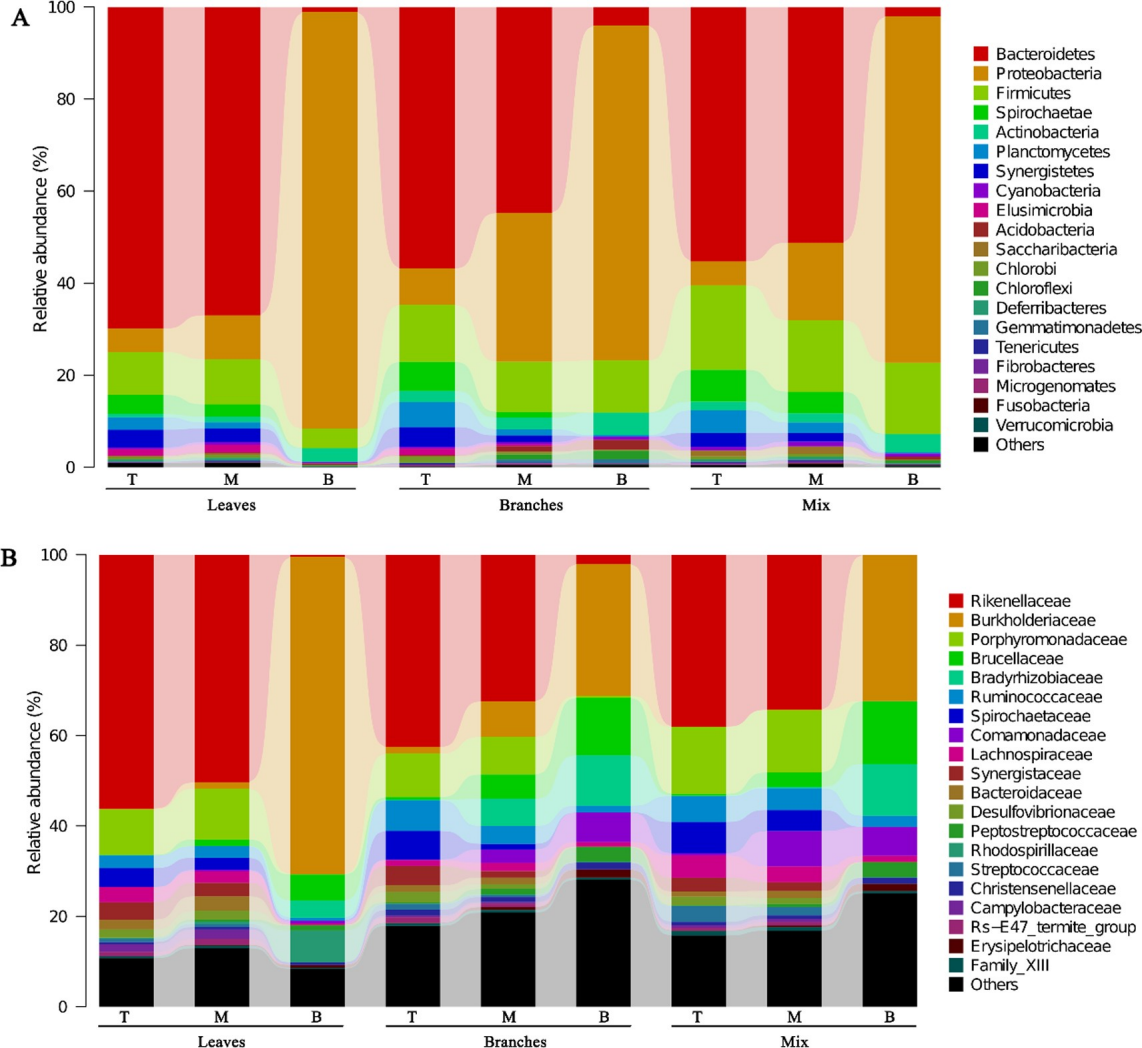
The bacterial community structure at different decomposition stages of fungus-combs. (A) The bacterial community structure at the phyla level. (B) The community composition at the family level.

**Fig 5:**
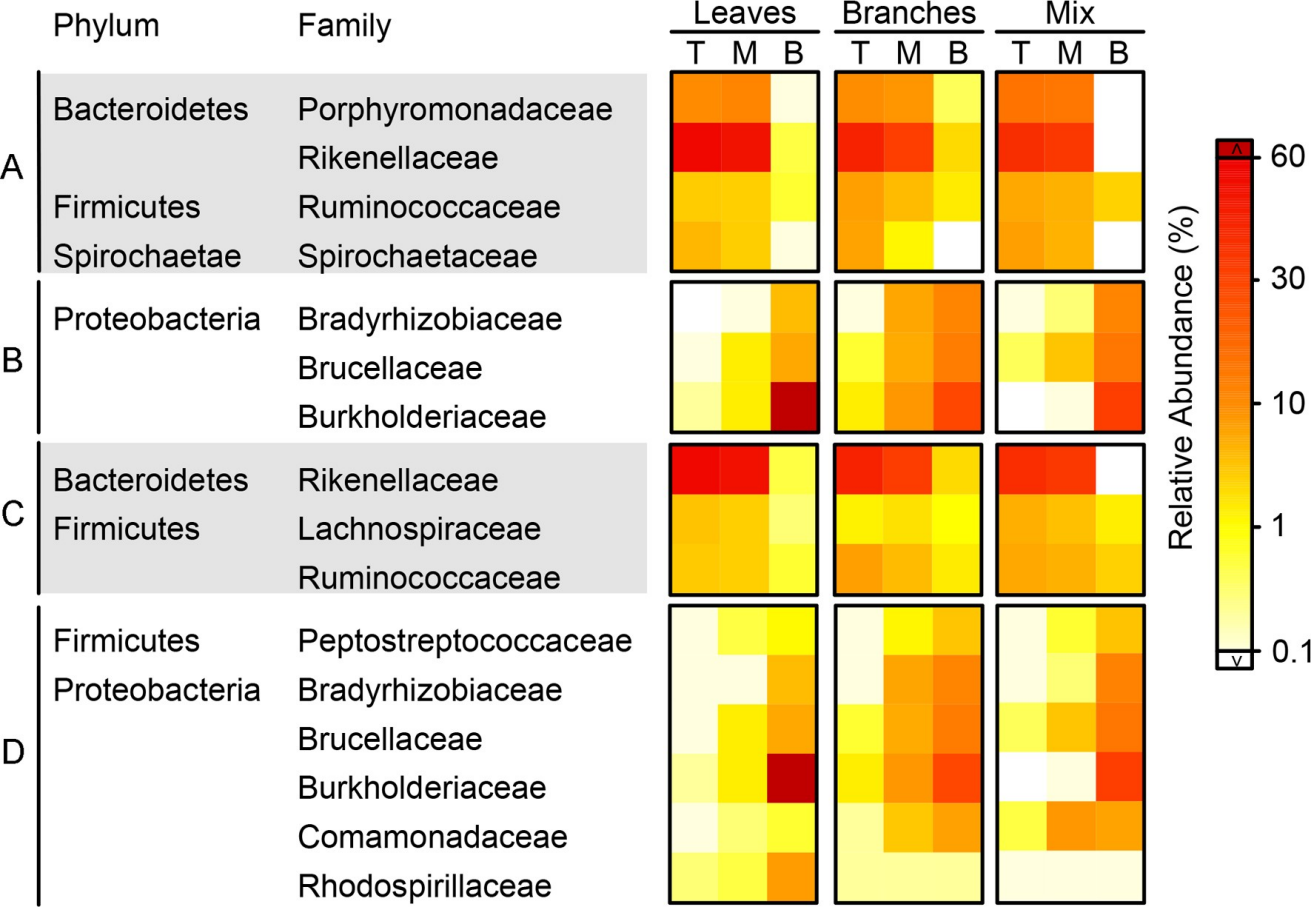
Heatmap of selected bacterial lineages at different decomposition stages of fungus-combs. There are four groups of bacterial lineages: A, enriched (RA > 5%) in fresh comb of three food treatments; B, enriched (RA > 5%) in mature comb of three food treatments; C, showed preference in fresh comb of specific food treatment(s); D, showed preference in mature comb of specific food treatment(s). T, M, and B described in Table 1.

At the phyla level, the most prominent first five out of seven bacterial groups comprised the members of Bacteroidetes, Proteobacteria, Firmicutes, Spirochaetae, Actinobacteria, Planctomycetes, and Acidobacteria (Fig 4A, for details, see S1 Table) were recorded in the top and middle of all combs. However, the relative abundances of Bacteroidetes, Planctomycetes, and Spirochaetes were reduced or even eliminated at the bottom of the combs, while Proteobacteria, Acidobacteria, and Actinobacteria were increased. The Firmicutes remained stable in all stages of the combs. At the family level, the abundances between the top and middle were similar but they were different from the bottom combs. There were also strong differences in the relative abundances of lineages among all fungus-combs across ages and food types (Figs 4B and 5). For example, Rhodospirillaceae and Burkholderiaceae preferred the bottom comb of oak leaves (BL), while Comaonadaceae preferred the bottom comb of oak branches and mix food (BB and BM). LEfSe analysis of fungus-combs across different decomposition stages indicated that specific bacterial groups may contribute to specific decomposition stage of the comb (S1 and S2 Figs). For instance, *Alistipes* was the most discriminatory genus in fresh combs, while *Burkholderia-Paraburkholderia* was the most discriminatory genus in mature combs. Meanwhile, a detailed taxonomic classification is attached to supplementary material (S2 Table).

### Effect of physicochemical indexes on bacterial community structure

To link the bacterial community with physicochemical indexes in combs, the constrained corresponding analysis (CCA) was performed (Fig 3C). The length of the lines with arrows represent the contribution of the corresponding factors to the variation in the bacterial community structure. The moisture content contributed the most followed by the organic matter and ash content. In case of diets, there was a positive interaction between bacterial diversity and organic matter (Tables 1 and 2, Fig 3C). Contrary to this, moisture and ash contents have a negative relation with the bacterial community. For example, moisture and ash contents were highest in leaves comb followed by mix food and branches. Opposite to this, organic matter and bacterial community were higher in branches followed by mix food and leaves. For comb stages, the organic matter had a strong positive interaction with bacterial diversity followed by moisture and ash contents. For example, among all combs, we generally recorded the highest bacterial diversity in the middle combs where organic matter was also high, but moisture and ash contents were not too high as compared to the bottom. On the basis of these results, we suggested that too high moisture and ash content, and low organic matter negatively affect the bacteria diversity.

## Discussion

Bacteria and the ectosymbiotic fungus (*Termitomyces*) share the habitat, fungus-comb [14–16]. *Termitomyces* produce various enzymes to degrade the plant material [10, 17, 19], while bacteria may contribute to decompose the biomass of fungus-comb in different pattern. Similar to termites, the leaf-cutting ants maintained fungus garden in their nests [44]. The biologists suggested that the functions of bacteria in the fungus garden of leaf-cutting ants are lignocellulose degradation, nitrogen fixation, nutrient supplementation, and antibiotic production [45–49], but whether these bacteria play similar roles in fungus-comb of termite, remains unclear. We present the detailed comparison of bacterial communities and physicochemical indexes among different decomposition stages in fungus-combs of termite (*O*. *formosanus*). We found that communities were similar among all combs but there were marked shifts in bacterial composition across comb ages over time. Moreover, the compositions of physicochemical indexes were different among all combs, as well as among all comb stages. Besides, bacterial diversity had a strong relationship with physicochemical indexes. Below, we discuss the consequences of these findings.

### The shift of dominant bacterial groups across the age-groups of combs

The dominant phyla and families were very similar at the same decomposition stages of all combs (S1 Table and Figs 4 and 5), may have a basic function of maintaining fungus-comb. To assign the role of bacteria in comb, we assumed that bacteria lineages enriched in the top comb may be shifted from termite gut and have complementary or similar roles in the comb as well as in the gut [15], while the community composition in the bottom comb may have a significant role only in a comb.

The dominant phyla in fungus-comb (see for details, S1 Table) are inconsistent with the previous studies on fungus cultivating termite combs and guts [15, 16, 50]. These phyla are important for carbohydrate metabolism, reductive acetogenesis, fungal cell wall degradation [10, 51, 52] and cello-oligomer degradation [53], which may help termite to get protein and fungal enzymes to cleave lignin [10]. As shown in S4 Table and Fig 5, the genera which were dominant in the “comb-age specific” bacterial families (Group A and B) and in “food-type specific” bacterial families (Group C and D) showed similar preferences with the corresponsive families for the same combs. For example, Rikenellaceae and its dominant genus, *Alistipes* both preferred the top combs. According to LEfSe analysis (S1 and S2 Figs), these genera were included all discriminatory genera across different aged combs by, except *Nitrospirillum* of Rhodospirillaceae which was not a discriminatory genus and it was only dominant in the bottom comb of leaf diet.

Porphyromonadaceae and Rikenellaceae are the most dominant families of Bacteroidetes in top comb, metabolize carbohydrates and produce organic acids like acetate, succinate, and butyrate [54, 55], to create an acidic environment for *Termitomyces* growth and better utilization of cellulose. *Alistipes* and *BCf9-17*, the members of Rikenellaceae, were the discriminatory genera in the top combs. These are gut‑specific bacterial lineages in fungus-cultivating termite [38, 50]. Furthermore, *Alistipes* has a positive correlation with protein diets [38, 50, 56], whereas, the functions of *BCf9-17* are poorly understood.

Spirochaetae and Planctomycetes were abundant both in top and middle comb, but they were scarce in bottom comb (S1 Table and Fig 4A). The traditional Planctomycetes are chemoorganoheterotrophs [57]. The discriminatory genus in top combs is an uncultured bacterium belong to an uncultivated group, vadinHA49 (S1 and S2 Figs), enriched in fungus-cultivating termite gut [58]. The specific functions of these microbes are out of the box. *Treponema* belonging to Spirochaetae was the discriminatory genus in the top combs (S2 Table). These bacteria are important for acetogenesis from H_2_ plus CO_2_ and nitrogen fixation in fungus-comb, however, it has been suggested that the abundance of nitrogen fixation bacteria is high in termite gut [59–64]. The potential of nitrogen fixation in fungus-comb requires further detailed investigation.

Proteobacteria and Actinobacteria were enriched in bottom comb, which is comparable with the previous works on fungus cultivating termite combs and guts [15, 65]. It has been suggested that Proteobacteria might assist in detoxification or food digestion [66–70], while Actinobacteria play a key role of defense through inhibiting the invasive fungus, *Pseudoxylaria* in fungus-cultivating termites [71]. The enrichment of Actinobacteria in mature comb might be from the surrounding soil by termites [65]. But, it is yet to be investigated that how are these bacteria regulated by termites? The genera *Nitrobacter*, *Ochrobactrum*, and *Burkholderia-Paraburkholderia* are the dominant members of Bradyrhizobiaceae, Brucellaceae, and Burkholderiaceae, respectively, belonging to Proteobacteria phyla, were discriminatory in bottom comb. It has been suggested previously that *Nitrobacter* are nitrite-oxidizing bacteria [72, 73]. *Ochrobactrum* microbes have lignolytic potential as well as these bacteria, also play a significant role in nitrite-oxidization [74–77]. *Burkholderia-Paraburkholderia* bacteria are capable to degrade aromatics [78].

### Preference of bacterial lineages to different diets

Bacterial lineages across different diets were compared at the family level (Fig 5). Rikenellaceae (Bacteroidetes) were high in the comb of leaves followed by branches and mix food combs. It has been suggested that Rikenellaceae metabolize carbohydrate actively [79]. *BCf9-17* genera of Rikenellaceae was abundantly higher in top comb of leaves compared to top combs of branches and mix food i.e., 10.40%, 0.70%, and 1.60% respectively (S4 Table). This genus is termite-specific, but its functions are poorly studied [65].

The specific family of Firmicutes showed its preference to specific diet. For example, Ruminococcaceae and Lachnospiraceae are important for cellulose degradation [80, 81], showed their preferences to different diets. Ruminococcaceae were relatively lower in leaves comb as compared to branches and mix food. While Lachnospiraceae were high in leaves and mix food comb. This is might be due to that Ruminococcaceae mainly degrade lignin rich in branch and mix food diets, while Lachnospiraceae utilize cellulose more accessible in leaves. Moreover, Peptostreptococcaceae of Firmicutes has potential to degrade hydrocarbons [82, 83], and these bacteria showed less preference to the comb of leaves (Fig 5). This is consistent with Schauer et al. [84] who described that a high-fiber diet caused shifts in the diversity of dominant gut bacteria in cockroach. Furthermore, the discriminatory genus in Peptostreptococcaceae, *Peptoclostridium*, has the ability to breakdown and utilize plant fibers [85, 86]. In bottom combs, the families of Proteobacteria also presented different preferences to different diets. Burkholderiaceae and Rhodospirillaceae were in favor of leaves, while Bradyrhizobiaceae, Brucellaceae, and Comamonadaceae preferred branches and mix food (Fig 5). Considering the capacity of Burkholderiaceae to degrade aromatic compounds [25, 87–90], their enrichment in leaves comb might deal with a higher content of aromatic compounds [61, 91, 92]. *Burkholderia-Paraburkholderia*, was the most enriched genus of Burkholderiaceae, has aromatics-degrading potential as we mentioned before [78]. Species of Rhodospirillaceae diverse in saline environment [93–95], may have the potential of halotolerance. The *Nitrospirillum* was contributed almost all abundance of Rhodospirillaceae and has a vital role in nitrification [96]. Recent research suggested that Comamonadaceae in forest soils can degrade lignin substrate [97]. *Delftia* was the most abundant genus of Comamonadaceae, and it has the capacity to degrade lignin-derived aromatic compounds [98]. Brucellaceae and Bradyrhizobiaceae are most important for nitrogen-fixation [99, 100]. *Ochrobactrum* and *Nitrobacter* were the most enriched genera of these families respectively, and their nitrite-oxidizing potential has been discussed before.

### The relationship of bacterial community and physicochemical indexes in the comb

The moisture content contributed the most to the variation in the bacterial community structure, followed by the organic matter and the ash content (Fig 3C). We recommend that specific amount of physicochemical indexes are required for better growth of bacterial diversity but excess moisture and ash contents, and low organic matter may harm their diversity (Fig 2 and Table 2). These findings are in the agreement with previous studies in soil [101–103]. Excess ash contents indicated the high salinity which can limit water availability [104] and it can also be toxic to metabolic activities [105]. Moisture can affect microbial activity by dissolving substrates [106]. Brockett et al. [107] reported that soil moisture and the potential lignocellulose-degrading activities of enzymes have a negative correlation. Thus, excess moisture could reduce the activities of the enzymes and lead to low nutrition availability in the comb. Soil organic matter content is often considered as a dominating factor of soil bacterial diversity [108, 109] because most soil micro-organisms rely on organic matter decomposition to obtain energy [110, 111]. Similarly, the effect of organic matter in comb can be explained by the availability of energy that most micro-organisms may obtain energy from organic matter in comb, and the low organic matter directly restricts the bacterial diversity and abundance.

## Conclusion

The current study gives a brief account of how bacterial community structure at different decomposition stages of fungus-comb respond to various lignocellulosic plant materials and physicochemical indexes. The shift of bacterial community across the fungus-comb over time indicated the adjustment of bacterial structure in the comb to deal with different kinds of diets (S3 Table, seeing the indicated functions). In addition, physicochemical indexes had been changed at different decomposition stages of comb and these also had a strong relationship with bacterial diversity. The extremely high moisture, low organic matter, and high ash content may reduce bacterial diversity. Furthermore, ruminants can only consume less lignified herbaceous plants such as soft grass [2]. Functional bacteria from the fungus-cultivating termites comb can be applied in the pretreatment of forestry and agricultural residues to improve their palatability and digestibility for ruminant animals.

## Supporting information

S1 Table Bacterial community structure at phyla-level across age-groups of different fungus-combs. (XLSX)

S2 Table A detailed taxonomic classification of bacteria groups. (XLSX)

S3 Table Indicated functions of predominant bacteria in fungus-combs. (XLSX)

S4 Table Selected bacterial lineages at different decomposition stages of different fungus-combs. (XLSX)

S5 Table NCBI accessions of samples. (XLSX)

S6 Table Denoising stats of cluster. (XLSX)

S1 Fig LDA scores observed for individual taxa that passed the LefSe significance threshold. Threshold on the logarithmic LDA score for discriminative features was 4.0.
(TIF)

S2 Fig Taxonomic cladogram of bacteria groups based on LDA scores. Only discriminant groups are presented. All datasets were submitted to the Sequence Read Archive of NCBI (http://www.ncbi.nlm.nih.gov; BioProject PRJNA604886).
(TIF)
