# Supplementary figures and images for: Exploring the effect of plant substrates on bacterial community structure in termite fungus-combs
Source: PLoS One. 2020 May 1;15(5):e0232329. doi: 10.1371/journal.pone.0232329 (PMC7194444; doi:10.1371/journal.pone.0232329)

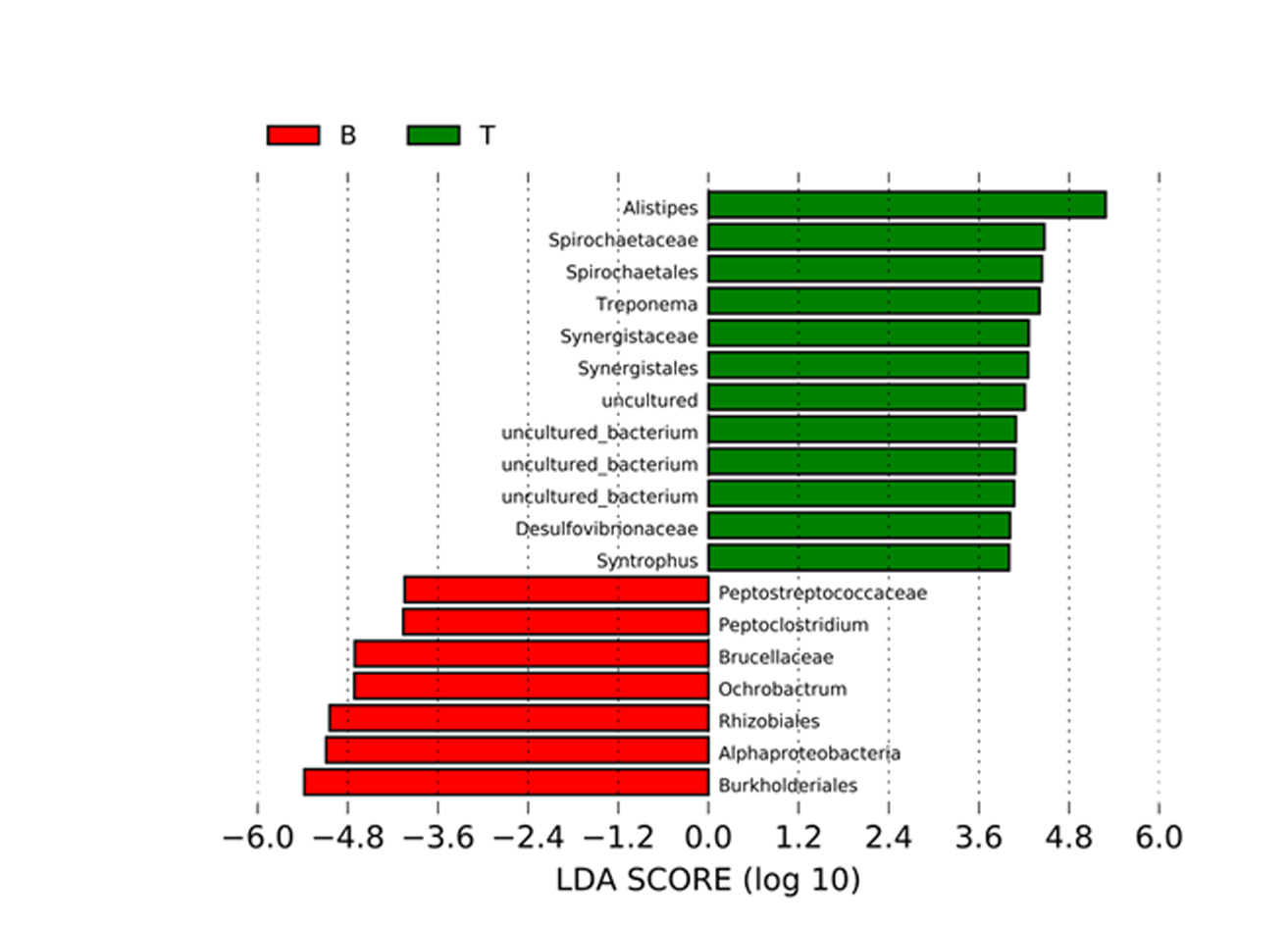

Supplement: S1 Fig — Threshold on the logarithmic LDA score for discriminative features was 4.0. (TIF) [file pone.0232329.s007.tif]

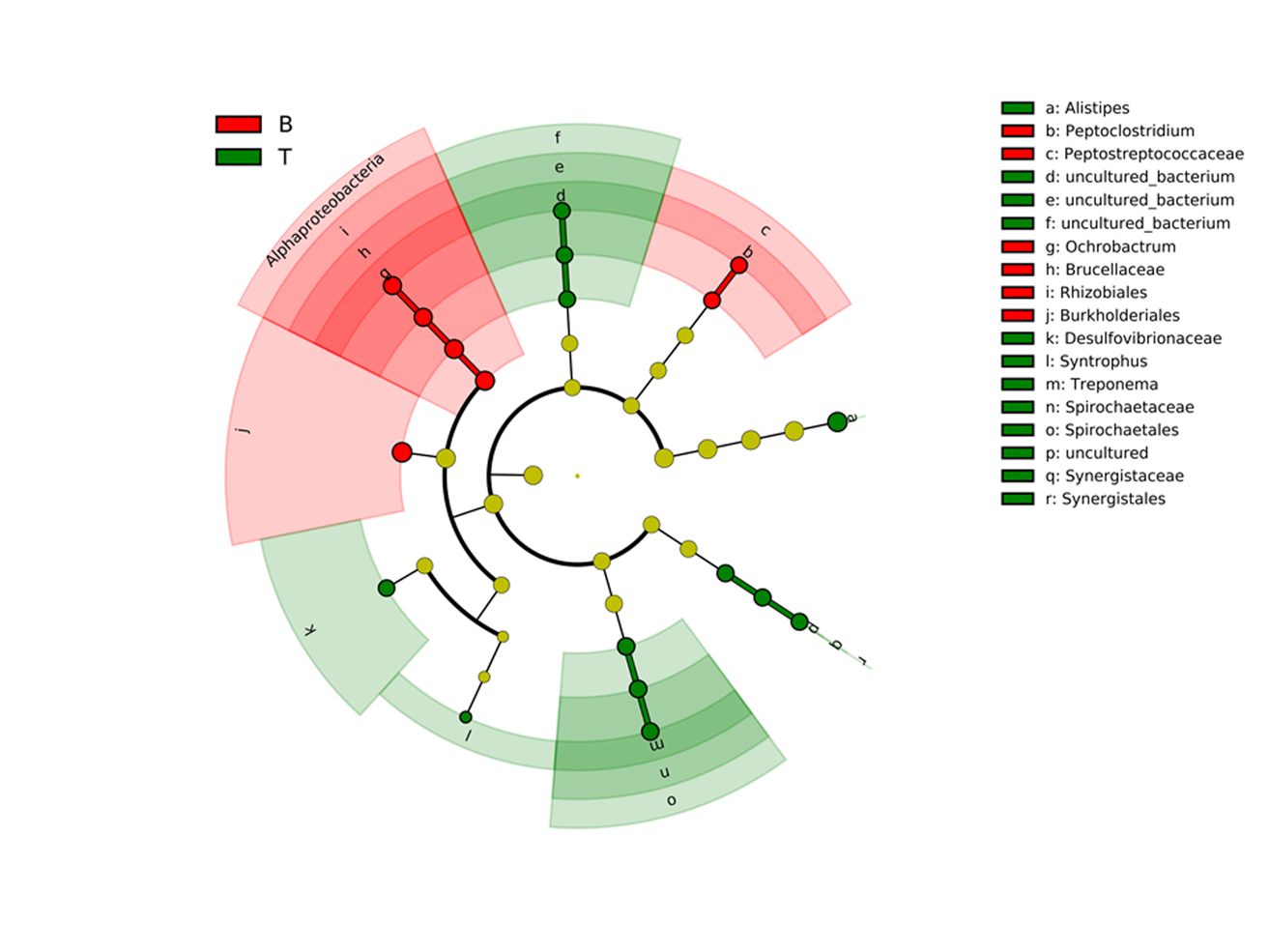

Supplement: S2 Fig — Only discriminant groups are presented. All datasets were submitted to the Sequence Read Archive of NCBI (http://www.ncbi.nlm.nih.gov; BioProject PRJNA604886). (TIF) [file pone.0232329.s008.tif]
